# The LOV Protein of *Xanthomonas citri* subsp. citri Plays a Significant Role in the Counteraction of Plant Immune Responses during Citrus Canker

**DOI:** 10.1371/journal.pone.0080930

**Published:** 2013-11-15

**Authors:** Ivana Kraiselburd, Lucas D. Daurelio, María Laura Tondo, Paz Merelo, Adriana A. Cortadi, Manuel Talón, Francisco R. Tadeo, Elena G. Orellano

**Affiliations:** 1 Instituto de Biología Molecular y Celular de Rosario (IBR - CONICET), Facultad de Ciencias Bioquímicas y Farmacéuticas (FBIOYF - UNR), Rosario, Santa Fe, Argentina; 2 Centre de Genómica, Institut Valencià d'Investigacions Agràries (IVIA), Montcada (València), Spain; 3 Área de Biología Vegetal, Facultad de Ciencias Bioquímicas y Farmacéuticas, Universidad Nacional de Rosario (UNR), Rosario, Santa Fe, Argentina; Beijing Institute of Microbiology and Epidemiology, China

## Abstract

Pathogens interaction with a host plant starts a set of immune responses that result in complex changes in gene expression and plant physiology. Light is an important modulator of plant defense response and recent studies have evidenced the novel influence of this environmental stimulus in the virulence of several bacterial pathogens. *Xanthomonas citri* subsp. citri is the bacterium responsible for citrus canker disease, which affects most citrus cultivars. The ability of this bacterium to colonize host plants is influenced by bacterial blue-light sensing through a LOV-domain protein and disease symptoms are considerably altered upon deletion of this protein. In this work we aimed to unravel the role of this photoreceptor during the bacterial counteraction of plant immune responses leading to citrus canker development. We performed a transcriptomic analysis in *Citrus sinensis* leaves inoculated with the wild type *X. citri* subsp. citri and with a mutant strain lacking the LOV protein by a cDNA microarray and evaluated the differentially regulated genes corresponding to specific biological processes. A down-regulation of photosynthesis-related genes (together with a corresponding decrease in photosynthesis rates) was observed upon bacterial infection, this effect being more pronounced in plants infected with the *lov*-mutant bacterial strain. Infection with this strain was also accompanied with the up-regulation of several secondary metabolism- and defense response-related genes. Moreover, we found that relevant plant physiological alterations triggered by pathogen attack such as cell wall fortification and tissue disruption were amplified during the *lov-*mutant strain infection. These results suggest the participation of the LOV-domain protein from *X. citri* subsp. citri in the bacterial counteraction of host plant defense response, contributing in this way to disease development.

## Introduction

During plant-pathogen interactions, plants first recognize pathogen-associated molecular patterns (PAMPs) which are slowly evolving molecular structures unique to microbes, such as bacterial flagellin or fungal chitin [1]. PAMPs recognition through specific receptors triggers the first line of plant innate immune responses or PAMP-triggered immunity (PTI). This response includes morphological and biochemical changes in the host aiming to create an unfavorable environment for the pathogen and prevent its establishment. Phytopathogens have evolved virulence effector proteins that modulate PTI and enable a successful infection [2]. These effectors are generally transferred into host cells via a type III secretion system. During the plant-pathogen “arms race” some plants have acquired resistance proteins to directly or indirectly recognize pathogen virulence effectors and trigger a second defense response. This effector-triggered immunity (ETI) is an accelerated and amplified version of PTI that suppresses bacterial growth conferring resistance to the disease. The ETI generally leads to a programmed cell death process in the site of infection, known as hypersensitive response (HR) [3]. During defense response, plants undergo physical changes such as cell wall thickening or callose deposition; biochemical modifications such as production of reactive oxygen species (ROS) and signaling compounds (salicylic acid, jasmonic acid, abscisic acid and ethylene) and synthesis of defense-related proteins and secondary metabolites such as phytoalexins, which prevent pathogen growth [4,5]. This complex set of responses involves a massive reprogramming of gene expression in the infected plant [6]. In spite of the high energy demand for the onset of immune responses and the biosynthesis of protective compounds, it has been shown that photosynthesis becomes down-regulated in the infected sites and that plants shift towards a non-assimilatory metabolism in response to several pathogens [7-9]. The reallocation of carbon metabolism favors the production of secondary compounds with antimicrobial activity, prioritizing plant defense responses, while down-regulation of photosynthesis restricts carbon source availability for the pathogen [7]. 

Light controls growth, development and behavior in living organisms including plants and microorganisms. There is growing evidence indicating that light also modulates defense responses and that an adequate light environment is required for plant resistance to a number of microbial pathogens [10]. Regarding bacteria, light is an important environmental factor not only for photosynthetic bacteria that use it as an energy source, but also for heterotrophic species. Recent reports demonstrated the influence of light in bacterial stress responses and in lifestyle transitions from motile/single-cell to surface-attached/multicellular states and from environmental to host-associated states [11-13]. With respect to pathogenic bacteria, light modulates virulence in many animal and plant pathogens including *Brucella abortus* [14], *Acinetobacter baumanni* [15] and *Xanthomonas citri* subsp. citri [16]. Light perception is carried out by biological photoreceptors that sense the wavelength and intensity of light and transduce this information into cellular pathways through a variety of signaling domains [17]. LOV domains are small blue-light sensing protein modules that belong to the PAS (Per-Arnt-Sim) superfamily [18]. These domains are normally associated to flavin mononucleotide (FMN) as a non-covalently bound chromophore. Blue-light absorption by this molecule triggers a photocycle that involves the reversible formation of a covalent bond between the 4a carbon of FMN and the thiol group of a conserved cysteine located in the LOV domain, rendering the active (signaling) state of the photoreceptor. In the absence of light it thermally converts to the dark, non-covalently bound, state of the protein [19]. LOV-domain proteins have been found in a large number of evolutionally diverse organisms belonging to all kingdoms [20]. 

*X. citri* subsp. citri is a Gram negative, gamma proteobacterium responsible for citrus canker, a severe disease that affects most commercial citrus cultivars causing significant crop losses worldwide. The pathogen enters host plant tissues through stomata and wounds and colonizes the apoplast, causing localized raised corky lesions on leaf, stem and fruit surfaces [21,22]. The genome of this bacterium has been completely sequenced [23]. It includes a gene coding for a LOV-domain protein. This protein, named Xcc-LOV, consists of an N-terminal LOV domain, associated to a histidine kinase and a response regulator domain located at the C-terminal end of the protein. In a recent work, we have reported that Xcc-LOV is a legitimate blue-light receptor with a typical LOV-type photochemistry and that light sensing through this photoreceptor is involved in the regulation of physiological processes in *X. citri* subsp. citri, directly associated with the bacterial ability to colonize host plants. Moreover, the disease symptoms in host plants infected with *X. citri* subsp. citri present a clear phenotypic variation by the deletion of this photoreceptor, indicating the participation of Xcc-LOV protein in the modulation of bacterial virulence [16]. The aim of this work was to investigate the role of the Xcc-LOV protein in the bacterial triggering of plant defense response. We performed a transcriptomic analysis of *Citrus sinensis* (sweet orange) leaves infected with the wild type (WT) *X. citri* subsp. citri and a mutant strain lacking the Xcc-LOV protein (Δ*lov* strain). The analysis revealed sets of genes corresponding to different biological processes, whose expression was significantly affected upon bacterial infection. These processes included photosynthesis, sucrose catabolism, isoprenoid and phenylpropanoid biosynthesis, cell wall and lipids metabolism, and biotic stress. We also evaluated physiological changes in plants inoculated with both strains of *X. citri* subsp. citri and observed that the Xcc-LOV protein mutant strain produced a stronger decrease in host photosynthesis, as well as higher tissue disruption in the site of infection than the WT strain. Moreover, other plant immunity-related alterations, such as cell wall reinforcement, were differently affected upon infection with the two *X. citri* subsp. citri strains, indicating a role of the Xcc-LOV protein in the bacterial counteraction of plant defense response, which favors the development of citrus canker disease.

## Results

### Infection with WT and Δ*lov* strains of *X. citri* subsp. citri leads to differential transcriptomic variations in *C. sinensis*

**Figure 1:**
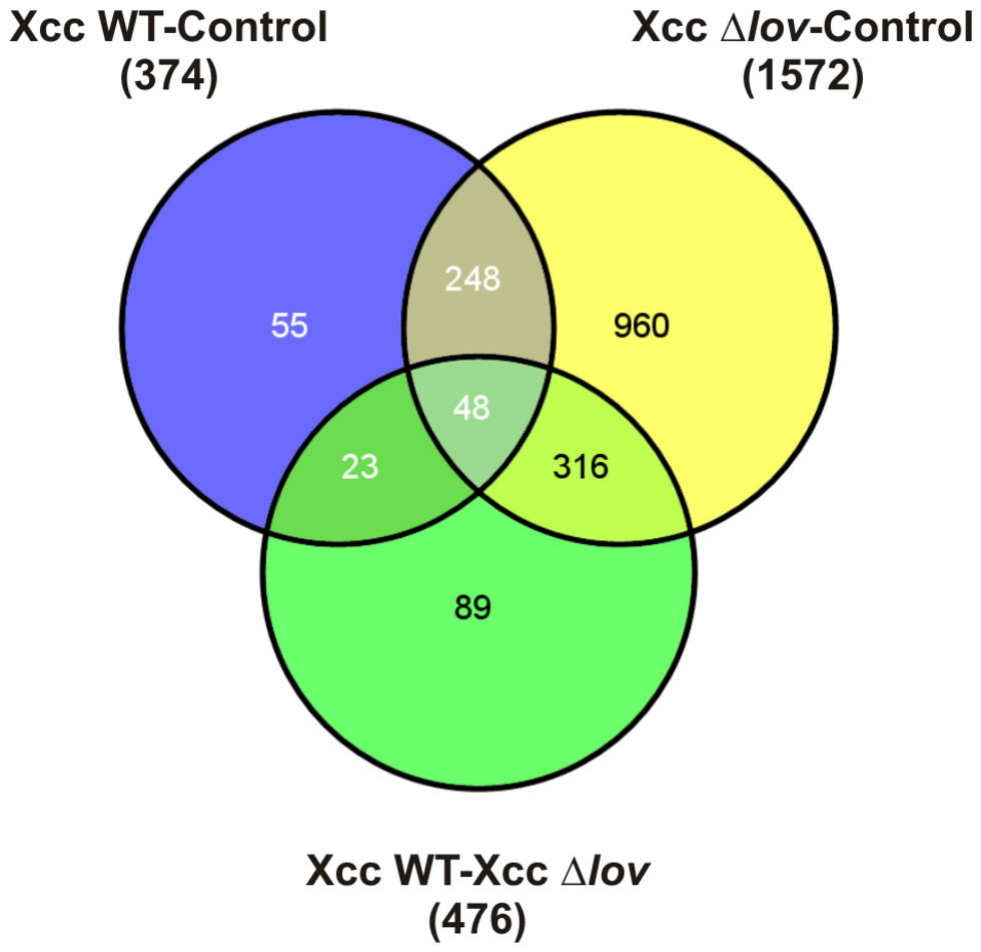
Transcriptomic analysis in orange leaves inoculated with *Xanthomonas citri* subsp. citri. *Citrus* cDNA microarray hybridization was performed 24 h after inoculation of orange leaves with WT and Δ*lov* strains of *X. citri* subsp. citri and control treatment (10 mM MgCl_2_). Three independents biological samples were used. Venn diagrams show the classification of differentially expressed microarray probes according to the following comparisons: *X. citri* subsp. citri WT-control, *X. citri* subsp. citri Δ*lov*-control and *X. citri* subsp. citri WT- *X. citri* subsp. citri Δlov.

We performed a transcriptomic analysis of orange leaves inoculated with WT and Δ*lov* strains of *X. citri* subsp. citri by cDNA microarray hybridization. The analysis was performed in leaves collected 24 h after bacterial and control treatments. Following microarray hybridization and scanning, as described in Materials and Methods, we selected as significant those microarray probes with an induction or repression of 2-fold relative to the other condition and with a maximum p-value (considering the FDR correction) of 0.01. These probes were classified into subgroups according to their differential expressions in the following comparisons: WT-control, Δ*lov*-control and WT-Δ*lov* (Figure 1). A total of 2422 probes (11.5 % of the probes present in the cDNA microarray) changed their expression considering both bacterial treatments. In order to evaluate plant genes specifically influenced by the presence of the LOV protein, we paid special attention to those probes that were differentially expressed in leaves inoculated with the Δ*lov* strain compared to the WT strain (476 probes). The sequences corresponding to the selected differentially expressed probes were related to their *Arabidopsis* orthologs and grouped into functional categories based on a gene ontology analysis using the agriGO web-tool and database and the MapMan software (Figure S1 and Figure S2) [24,25]. Table 1 shows the over-representation analysis of differentially expressed gene families during the interaction of orange leaves with *X. citri* subsp. citri WT and Δ*lov* mutant, for genes with M > 1 and M < -1, where M is log_2_[expression for WT treatment/expression for Δ*lov* treatment]. The over-represented functional categories included photosynthesis, sucrose and starch catabolism, secondary metabolism, lipid metabolism, cell wall modifications and defense response. 

**Table 1:** Over-representation analysis of differentially expressed gene families during the orange interaction with *Xanthomonas citri* subsp. citri WT and Δ*lov* mutant for genes with M > 1 (A) and M < -1 (B), where M is log_2_[expression for WT treatment/expression for Δ*lov* treatment].

**A**					
**GO term**	**Description**	**Number in input list**	**Number in reference**	**p-value**	**FDR**
GO:0015979	photosynthesis	15	85	2.7e-12	1.8e-10
GO:0019684	photosynthesis, light reaction	8	46	4.6e-07	1.5e-05
GO:0006091	generation of precursor metabolites	11	128	2.0e-06	4.5e-05
**B**					
**GO term**	**Description**	**Number in input list**	**Number in reference**	**p-value**	**FDR**
GO:0009814	defense response, incompatible interaction	9	52	1.1e-05	0.0016
GO:0044283	small molecule biosynthetic process	21	337	0.00011	0.003
GO:0002376	immune system process	11	110	0.00013	0.003
GO:0006955	immune response	11	109	0.00012	0.003
GO:0009816	defense response to bacterium	5	15	8.0e-05	0.003
GO:0045087	innate immune response	11	99	5.3e-05	0.003
GO:0044255	cellular lipid metabolic process	16	225	0.00018	0.0036
GO:0044281	small molecule metabolic process	34	742	0.00029	0.0047
GO:0032787	monocarboxylic acid metabolic process	14	187	0.00028	0.0047
GO:0019438	aromatic compound biosynthetic process	10	107	0.00042	0.006
GO:0006725	aromatic compound metabolic process	13	174	0.00046	0.006
GO:0043436	oxoacid metabolic process	21	391	0.00071	0.0061
GO:0006952	defense response	15	230	0.00067	0.0061
GO:0006950	response to stress	37	880	0.00077	0.0061
GO:0006631	fatty acid metabolic process	10	111	0.00055	0.0061
GO:0006082	organic acid metabolic process	21	392	0.00073	0.0061
GO:0019752	carboxylic acid metabolic process	21	391	0.00071	0.0061
GO:0008610	lipid biosynthetic process	13	183	0.00073	0.0061
GO:0042180	cellular ketone metabolic process	21	401	0.00096	0.0072
GO:0009699	phenylpropanoid biosynthetic process	7	62	0.0011	0.0078
GO:0009611	response to wounding	8	81	0.0011	0.0078
GO:0042221	response to chemical stimulus	33	786	0.0015	0.01

The number of genes with modified expression during the response (input list), the number of genes represented in the microarray (reference), p-value for the respective analysis and false discovery rate correction (FDR) are indicated for each Gene Ontology category (GO term), considering a cut-off of FDR <0.01.

### *X. citri* subsp. citri *Δlov* strain causes more pronounced photosynthesis down-regulation in *C. sinensis* leaves than the WT strain and induces metabolic reallocation- and defense response-related gene expression

We found differential expression of 25 microarray probes corresponding to photosynthesis-related genes, all of which were down-regulated in Δ*lov-*inoculated orange leaves, compared to WT *X. citri* subsp. citri-inoculated leaves. Of these, 9 genes showed homology with chlorophyll-binding proteins belonging to the light harvesting complexes associated with photosystems (PS) I and II (LHC I, LHC II), 13 were homologous to subunits of PSI and PSII, 1 to a subunit of the cytochrome b6/f complex and 2 to other proteins involved in electron transfer: plastocyanin and ferredoxin-NADP^+^-oxidoreductase (Table S1, category Photosynthesis). Microarray expressions of a PSII reaction centre protein and of plastocyanin 1 were confirmed by real-time RT-PCR analysis (Figure S3). Moreover, a light-harvesting chlorophyll B-binding protein 3 and a protein from the LHC II were represented by three and two different probes of the microarray, respectively, with similar expression ratios. This fact further supported the reproducibility of microarrays results. The products of the mentioned genes are directly involved in the photosynthetic process of the host plant, indicating that this process would be affected in plants exposed to the bacterial treatment, and that this effect would be different in *X. citri* subsp. citri WT- and Δ*lov*-treated plants. 

**Figure 2:**
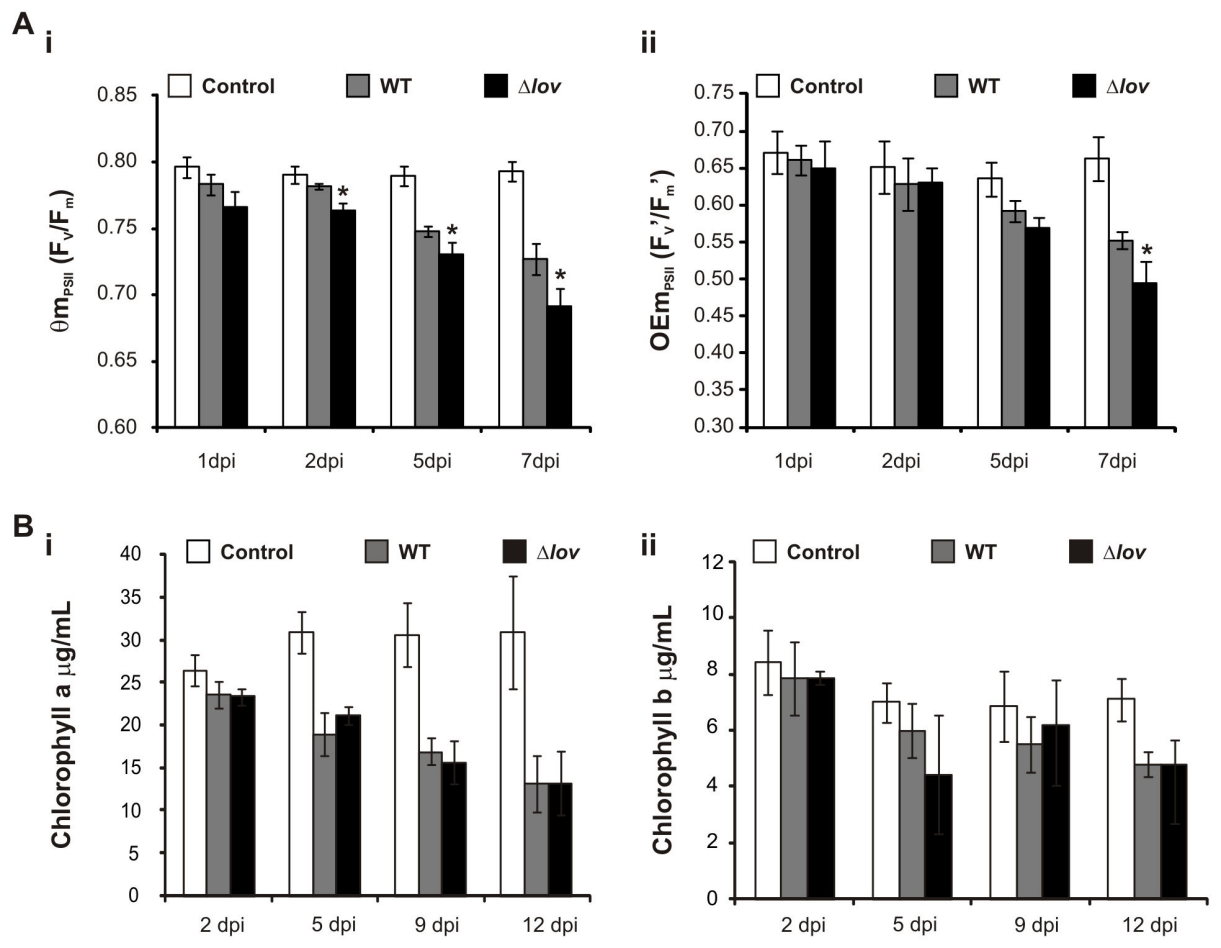
Evaluation of host photosynthesis during *Xanthomonas citri* subsp. citri interaction with orange leaves. (**A**) Chlorophyll fluorescence parameters were calculated in orange leaves inoculated with the WT and Δ*lov* *X. citri* subsp. citri strains and control treatments (10 mM MgCl_2_) at different days post inoculation (dpi): Φm_PSII_ corresponds to maximum quantum yield of PSII in the dark adapted state (F_v_/F_m_, **Ai**), OEm_PSII_ corresponds to maximum operating efficiency of PSII (F’_v_ /F’_m_, **Aii**). F_m_: maximum chlorophyll fluorescence in dark adapted leaves; F_v_: variable fluorescence in dark adapted leaves; F_m_’: maximum chlorophyll fluorescence after saturating light pulses, F_v_’: variable fluorescence after saturating light pulses. (**B**) Chlorophyll a (**i**) and b (ii) contents were measured at different times after orange inoculation with *X. citri* subsp. citri WT and Δ*lov* strains and control treatment. All results are expressed as the mean of three independent biological replicates and error bars represent the standard deviations. Asterisks indicate significant differences between WT and Δ*lov* treatments (p<0.05).

In order to evaluate the photosynthetic performance in host plants, we carried out chlorophyll fluorescence measurements in orange leaves inoculated with *X. citri* subsp. citri WT and Δ*lov* strains and in control leaves at different times after inoculation, to calculate the photosynthetic parameters: maximum quantum yield of PSII (Φm_PSII_) and maximum operating efficiency of PSII (OEm_PSII_). ΦmPSII represents the maximum photochemical efficiency of PSII, whereas OEmPSII is the efficiency with which the light absorbed by chlorophylls associated with PSII is used for the photochemical reduction of the primary-acceptor when all centers are oxidized. We could observe that these parameters suffered an important reduction on inoculated leaves compared to control leaves (Figure 2A). A decrease in these parameters, particularly in Φm_PSII_ is frequently observed in plants exposed to abiotic and biotic stresses. In our results, the observed reduction of Φm_PSII_ and OEm_PSII_ is consistent with the expected effects of the pathogen’s infection. However, we could observe a more marked reduction of these parameters in leaves inoculated with the Δ*lov* strain, compared to those inoculated with the WT strain (Figure 2A). These results indicate that photosynthesis is more severely affected in leaves inoculated with the *X. citri* subsp. citri Δlov strain. 

**Figure 3:**
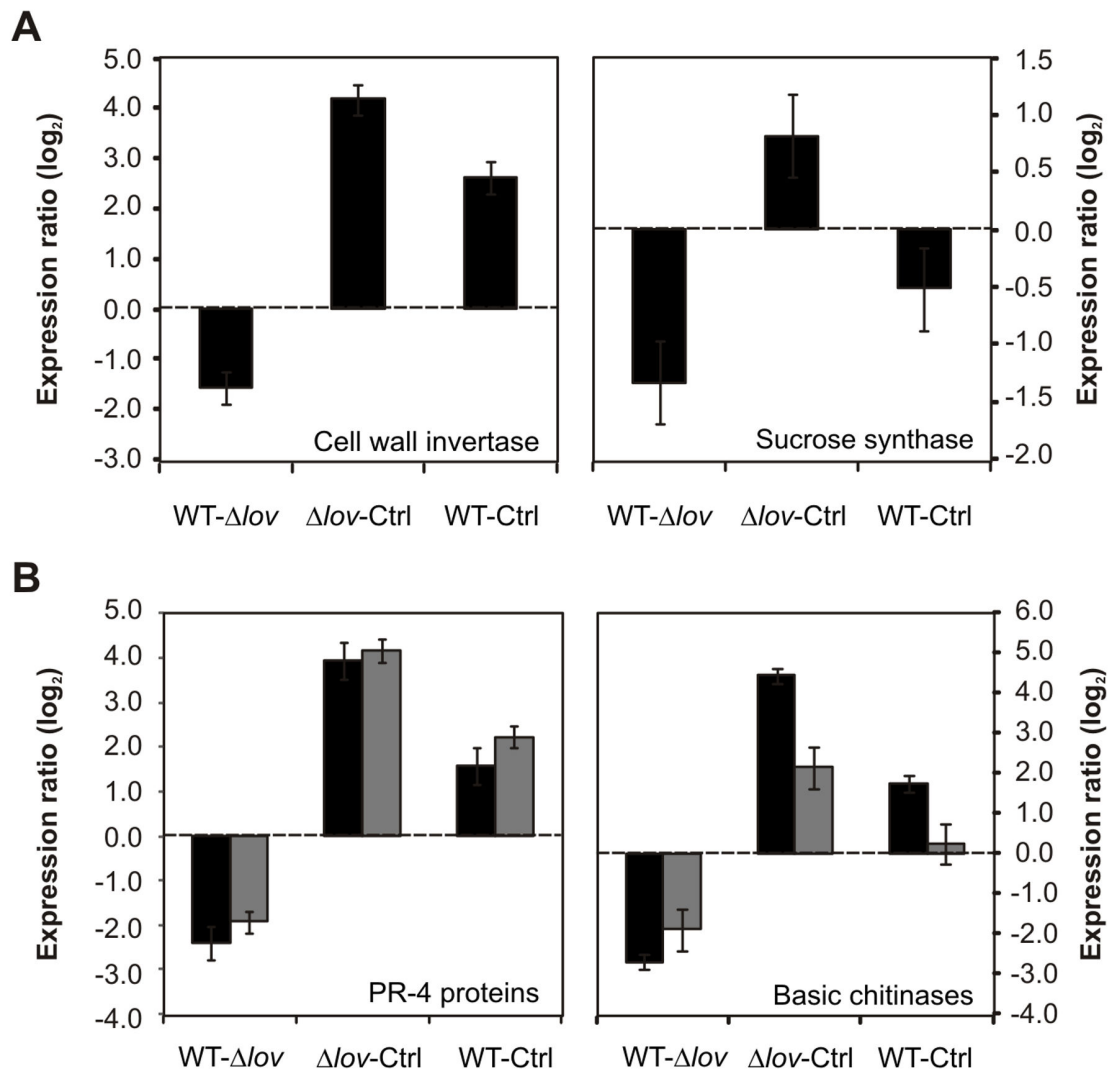
CHO metabolism- and defense response-related *Citrus* ESTs differentially regulated during *Xanthomonas citri* subsp. citri interaction. Expression ratios were calculated for genes differentially expressed in orange leaves inoculated with the WT and Δ*lov* *X. citri* subsp. citri strains and control treatments (10 mM MgCl_2_, Ctrl) (**A**) Log_2_ of expression ratio between treatments (M) for ESTs corresponding to a cell wall invertase and a sucrose synthase (orange1.1g008242m and orange1.1g008531m of *Citrus sinensis*) (**B**) Log_2_ of expression ratio between treatments (M) for ESTs corresponding to two pathogenesis-related proteins [orange1.1g032389m (black columns) and orange1.1g032285m (grey columns) of *C. sinensis*] and two chitinases [orange1.1g020187m (black columns) and orange1.1g026315m (grey columns) of *C. sinensis*]. Data are averages of values obtained from three independent biological samples. Bars represent standard error. Accession numbers correspond to the complete *C. sinensis* genes taken from the phytozome database (http://www.phytozome.net/citrus.php).

In order to evaluate potential changes in plant chlorophylls, we quantified chlorophyll a and b contents in *X. citri* subsp. citri WT- and Δ*lov*-inoculated orange leaves and in control leaves at different times after inoculation. While we could observe a gradual reduction of both pigments in leaves exposed to bacterial treatment compared with control leaves, no significant differences were observed in chlorophyll content between leaves inoculated with the different *X. citri* subsp. citri strains (Figure 2B). These results indicate that the down-regulation of photosynthesis observed in leaves inoculated with the Δ*lov* strain is not correlated with variations in pigment contents. A decrease in photosynthesis rate is a widespread pattern during plant-pathogen interactions, and it is normally accompanied with a “source to sink transition” in the infected tissues. During this transition, the stimulation of sucrose degradation processes is generally observed, particularly by the induction of expression and activity of plant invertases [7]. In our transcriptomic analysis we could identify the up-regulation of 6 *Citrus* probes corresponding to enzymes involved in sucrose and starch catabolism upon the treatment with *X. citri* subsp. citri Δlov strain (Table S1, category CHO Metabolism). Of these probes, 2 correspond to enzymes with invertase activity (cell wall invertases) and 1 to a sucrose synthase enzyme (also involved in sucrose cleavage). Figure 3A shows the log_2_ of the expression ratio between treatments of *citrus* ESTs corresponding to a cell wall invertase and a sucrose synthase. The increased expression of these genes would be consistent with a metabolic transition in infected plant tissues, this effect being more pronounced in *X. citri* subsp. citri Δ*lov*-inoculated leaves. In several plant-pathogen interactions, this metabolic transition is related to an increased expression of defense-related genes [7]. Accordingly, we detected the up-regulation of 4 microarray probes corresponding to defense response-related genes upon inoculation of orange leaves with the Δ*lov* strain of *X. citri* subsp. citri (Table S1, category Defense Response). They include 2 basic chitinases and 2 pathogenesis related (PR)-4 proteins. Figure 3B shows the log_2_ of the expression ratio between treatments for these genes. Microarray expression of a cell wall invertase, a basic chitinase and a PR-4 protein were confirmed by real-time RT-PCR analysis (Figure S3).

### *X. citri* subsp. citri *Δlov* strain enhances secondary metabolism modifications and lignin deposition in *C. sinensis* leaves

**Figure 4:**
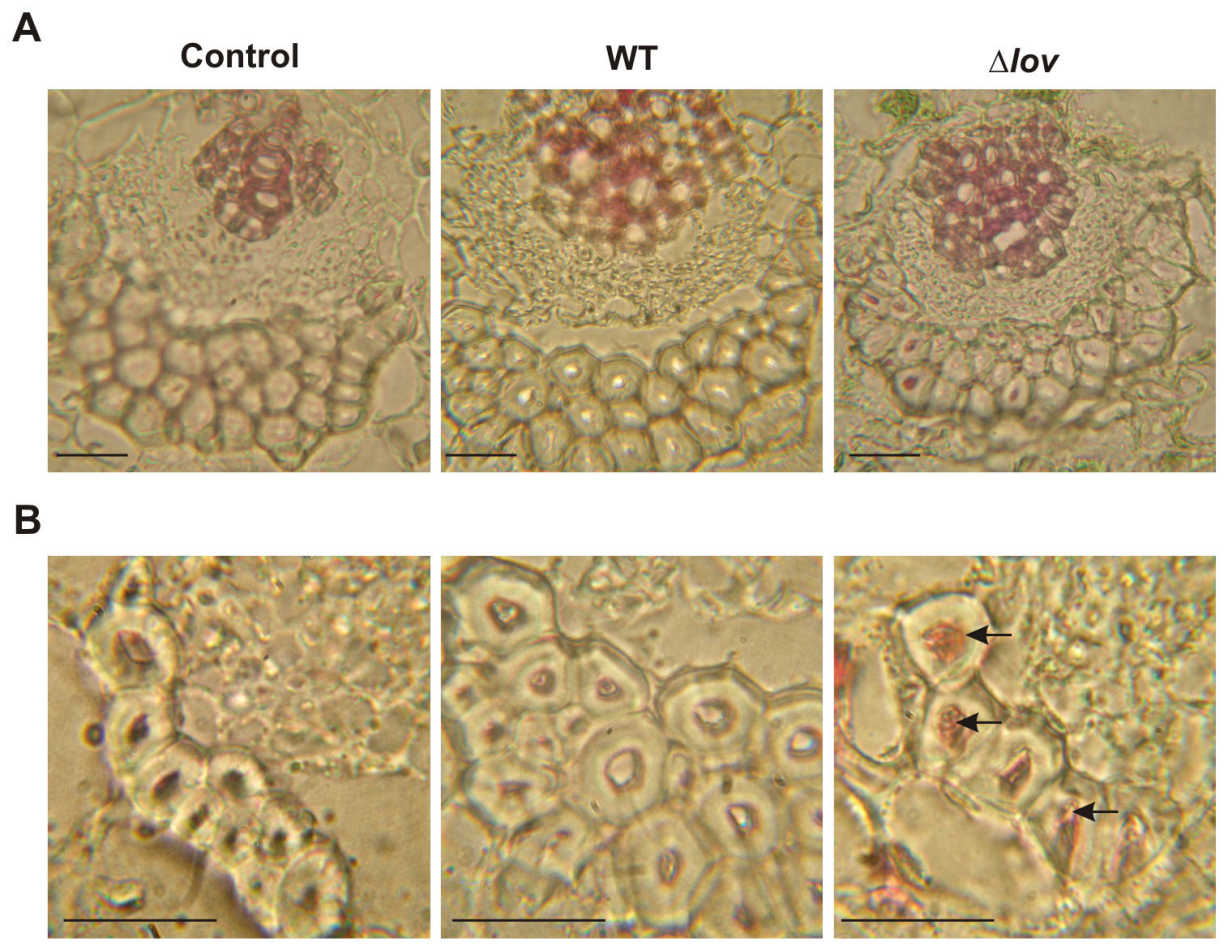
Histological analysis of lignin deposition in orange leaves upon interaction with *Xanthomonas citri* subsp. citri. Lignin deposition was analysed by acid fluoroglucin staining of orange leaves inoculated with WT and Δ*lov* *X. citri* subsp. citri strains and control treatments (10 mM MgCl_2_). Stained tissues were observed with a visible microscope at 7 days post inoculation (dpi) using a 1000X magnification. The wine-red coloration represents lignin deposition in plant secondary cell walls. **3A** panels show infected tissues sections in which xylem vessels as well as phloem and sclerenchymatous fibers surrounding the vascular bundles are visualized. **3B** panels show zoomed-in images of sclerenchymatous fibers where internal lignin content is indicated with arrows. Scale bars: 5 µm. Identical results were obtained with three independent biological samples.

We found differential expression of 21 microarray probes corresponding to genes involved in secondary metabolism; particularly 17 genes correspond to components of isoprenoids and phenylpropanoids biosynthesis pathways. Of the 21 probes, 20 resulted up-regulated in leaves treated with the Δ*lov* strain compared to those treated with the WT strain of *X. citri* subsp. citri and with control leaves (Table S1, category Secondary Metabolism). Isoprenoids and phenylpropanoids constitute a large group of compounds that fulfill different roles in plant development, physiology and defense against pathogens [26]. Phenylpropanoid molecules are the constituents of support structures such as lignin, which can act as inducible physical barriers against pathogen entrance and propagation [27,28]. We could observe that 8 lignin biosynthesis-related *Citrus* probes showed up-regulation upon plant treatment with the Δ*lov* strain of *X. citri* subsp. citri. These probes correspond to genes coding for the enzymes phenylalanine ammonia-lyase (PAL) 1, elicitor-activated gene 3-2, 2 enzymes with hydroxycinnamoyl transferase activity, 1 enzyme of the S-adenosyl-L-methionine-dependent methyltransferases superfamily, and 2 enzymes with O-methyltransferase activity. Microarray expression of PAL 1 was confirmed by real-time RT-PCR analysis (Figure S3). Moreover, a hydroxycinnamoyl-CoA shikimate/quinate hydroxycinnamoyl transferase gene was represented by two different probes of the microarray with similar expression ratios (Table S1). PAL catalyzes the first step of lignin biosynthesis and is known to be induced by exposure to microbial pathogens [29,30]. The increased expression of this protein in leaves inoculated with the Δ*lov* strain of *X. citri* subsp. citri suggests a higher lignification of plant secondary walls in these leaves. In order to evaluate lignin content we performed an acid fluoruglucin staining in inoculated orange leaves. This method allows the visualization of lignin contents in plant tissues as lignified cell walls acquire a wine-red color. Figure 4 shows the visual microscopic observation of orange tissues 7 days after bacterial and control inoculation. For all the treatments, we could observe a characteristic lignin staining around the xylem vessels and on the sclerenchymatous fibers surrounding the vascular bundles. However, lignin distribution in the host tissues exhibited significant differences between treatments. While in control leaves lignin distribution remained mainly around xylem vessels, in leaves treated with both *X. citri* subsp. citri strains an important lignin accumulation was also observable in the sclerenchymatous fibers. Noteworthy, in leaves inoculated with the Δ*lov* strain, lignin content was highly concentrated in the cellular lumen of these fibers where a more intense coloration was observed. This result is indicative of an increased lignin deposition in the *X. citri* subsp. citri Δ*lov*-inoculated leaves.

### *X. citri* subsp. citri *Δlov* strain causes more pronounced alterations in host tissue integrity than the WT strain in *C. sinensis* leaves

**Figure 5:**
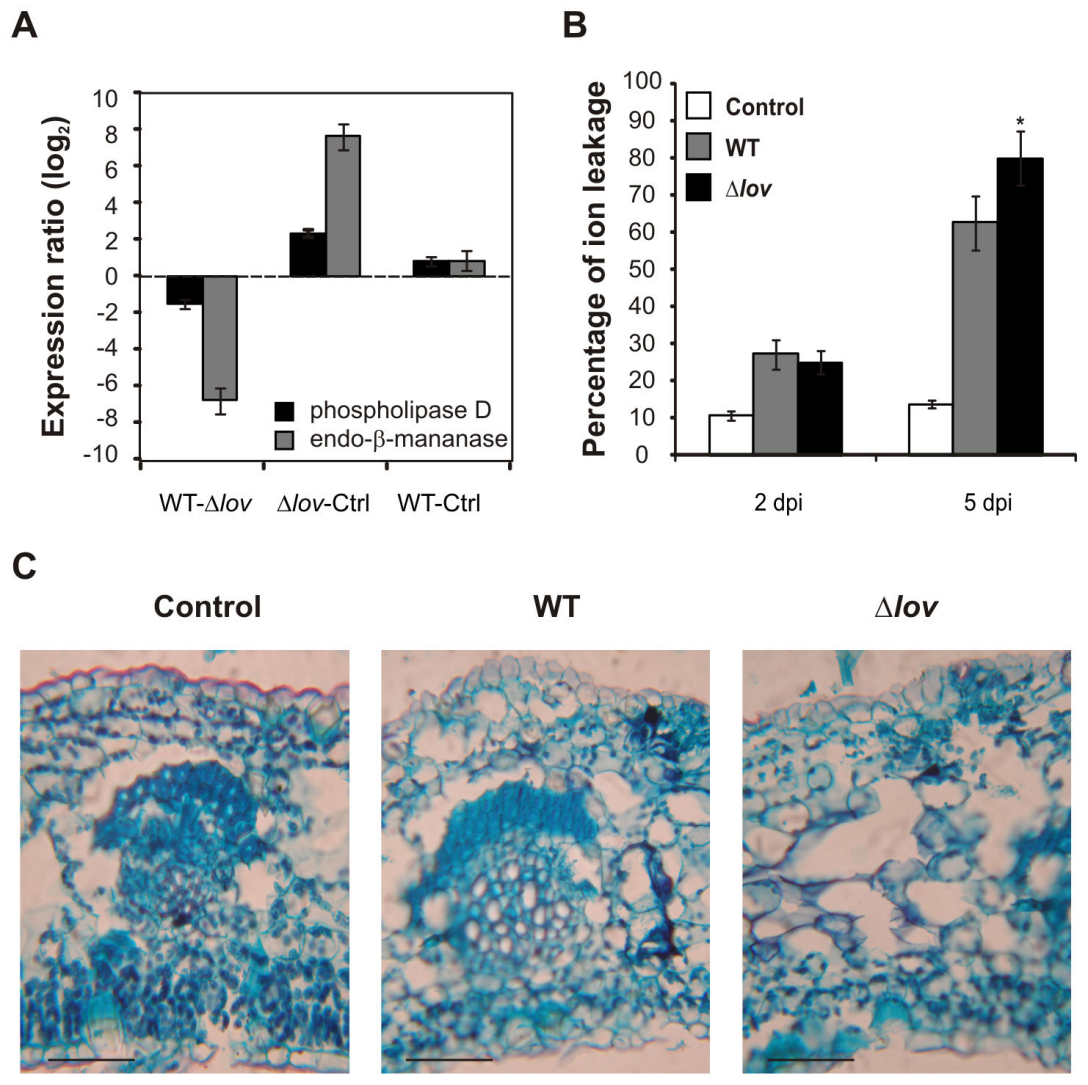
Tissue integrity upon orange interaction with WT and Δ*lov* strains of *Xanthomonas citri* subsp. citri. (**A**) Analysis of log_2_ of expression ratio between treatments (M) of a *Citrus* EST corresponding to a phospholipase D (orange1.1g003057m of *Citrus sinensis*) and an endo-β-mannanase (orange1.1g013811m of *C. sinensis*). Data are averages of values obtained from three independent biological samples. Bars represent standard error. Accession numbers correspond to the complete *C. sinensis* genes taken from the phytozome database (http://www.phytozome.net/citrus.php). Ctrl: control treatment (10 mM MgCl_2_) (**B**) Ion leakage measurements were performed in bacterial- and control (10 mM MgCl_2_)-inoculated orange leaves at 2 and 5 days post inoculation (dpi). Results are expressed as percentage of ion leakage and represent the mean of three independent biological replicates. Error bars represent the standard deviations. Asterisks indicate significant differences between WT and Δ*lov* treatments (p<0.05). (**C**) Structure and organization of inoculated-leaves fragments were analyzed by safranine/fast green staining. Panels show the microscopic visualization of stained tissue fragments 7 days after bacterial and control treatments using a 400X magnification. Scale bars: 10 µm. Identical results were obtained with three independent biological samples, 90% of the optical fields analyzed for each biological replica rendered the same results.

During the *Citrus* transcriptomic analysis we found the up-regulation of 13 microarray probes corresponding to genes encoding lipid metabolism-related proteins upon infection with the Δ*lov* strain of *X. citri* subsp. citri compared to leaves subjected to the WT strain and to control treatments (Table S1, category Lipid Metabolism). These genes encode enzymes involved in the synthesis, elongation and modification of fatty acids (1 plastidic pyruvate kinase β subunit 1, 1 protein of the AMP-dependent synthetases and ligases families and 1 fatty acid desaturase 2); glycolipid and phospholipids synthesis (1 digalactosyl diacylglycerol deficient 2 and 1 protein of the S-adenosyl-L-methionine-dependent methyltransferases superfamily); a lipid transfer protein, and several lipid degradation proteins (1 protein of the α/β-hydrolases superfamily, 1 phospholipase D β 1, 1 protein of the phospholipase C-like phosphodiesterases superfamily protein, 1 ATP-dependent caseinolytic proteases/crotonases family protein, 1 multifunctional protein and 1 acyl-CoA oxidase 4). Several of these genes were previously reported to act during plant immunity in different plant-pathogen interactions [31-33]. Noticeably, the phospholipase D β 1 was represented by two different microarray probes, showing an up-regulation upon orange inoculation with the Δ*lov* strain of *X. citri* subsp. citri (Figure 5A). Microarray expression for this protein and for a fatty acid desaturase 2 was confirmed by real-time RT-PCR analysis (Figure S3). 

The variation of lipid metabolism enzymes suggests a potential effect in the integrity of plant cell membranes upon treatment with the Δ*lov* strain of *X. citri* subsp. citri. As alterations in the integrity of cell membranes can produce changes in membrane permeability resulting in the release of electrolytes from the cells, the damage caused by stress can be quantified by measuring the electrical conductivity of plant tissues immersed in an aqueous solution (ion leakage assay, [34]). We performed ion leakage measurements in orange leaves inoculated with *X. citri* subsp. citri WT and Δ*lov* strains and in control leaves at different times after treatment. Two days after treatment, a similar increase on cell membranes permeability was observed in leaves inoculated with both bacterial strains. After five days, membrane permeabilities showed a further increase, however this effect was stronger in orange leaves inoculated with the Δ*lov* strain of *X. citri* subsp. citri (Figure 5B). This result indicates a more pronounced effect on plant tissue integrity upon infection with the mutant strain compared to the WT strain.

We also performed a histological staining of inoculated leaves fragments for the microscopic evaluation of plant tissues structure. In order to identify potential tissue disruption produced by the fixation and staining procedures, we obtained 5-10 serial sections of each biological replica. More than 90 % of the sections analyzed for each treatment revealed a conserved tissue damage pattern, indicating that almost no additional damage was generated during the preparation of the samples. We could observe a severe structure disorganization and mesophyll cell lysis in *X. citri* subsp. citri Δlov-treated tissues while no extensive damage was observed in WT-treated tissues (Figure 5C). This result is consistent with the higher ion permeability of the tissues inoculated with the Δ*lov* strain of *X. citri* subsp. citri. 

Additionally, we found differential expression of 8 microarray probes corresponding to genes encoding cell wall modification-related proteins. These include 1 leucine-rich repeat (LRR)/extensin family protein, 1 glycosyl hydrolases superfamily protein, 2 pectin lyase-like superfamily protein, 1 xyloglucan endotransglucosylase/hydrolase 5, 1 expansin-like B1 protein and 1 pectinacetylesterases family protein (Table S1, category Cell Wall). Particularly, the expression of a microarray probe corresponding to an endo-β-mannanase involved in cell wall degradation was highly up-regulated upon inoculation with the Δ*lov* strain of *X. citri* subsp. citri, further indicating integrity alterations in infected tissues (Figure 5A). 

The expression of a subset of the *Citrus* genes discussed in this work as well as plant physiological alterations have also been evaluated in orange leaves inoculated with a ∆*lov* strain complemented *in trans* with the wild type copy of the *lov* gene verifying the reversion of the WT phenotypes (detailed in Methods and Results S1 and in Figure S4).

## Discussion

Plants are constantly exposed to pathogens. However, since plants are resistant to most microorganisms, only a small proportion of these interactions results in successful pathogen spreading. During plant interactions with pathogens, recognition of the invading organism triggers the activation of a plethora of immune responses. These include closure of stomata to limit pathogen entry sites, fortification of the cell wall, production of ROS and synthesis of signaling compounds such as salicylic acid, defense-related proteins and secondary metabolites such as phytoalexins [7]. These complex plant responses are directed by a reprogramming of host gene expression in the presence of the pathogen. Depending on their lifestyle, plant pathogens can be classified as biotrophs, hemibiotrophs, and necrotrophs. Biotrophic and hemibiotrophic pathogens utilize plant-derived metabolites for growth and, therefore, aim to maintain the cellular integrity of the host plant. In the case of hemibiotrophic pathogens, host tissues are killed in the late stages of the infection. Necrotrophs, in contrast, induce necrosis to utilize the cellular components of the collapsing host tissue [35]. *X. citri* subsp. citri is a hemibiotrophic pathogen responsible for citrus canker, a devastating disease affecting the *Citrus* genus. In a previous report, we described the construction of a mutant *X. citri* subsp. citri strain called Δlov, lacking a LOV-type blue-light photoreceptor (Xcc-LOV protein). This photoreceptor participates in the regulation of multiple physiological processes directly associated with the pathogen ability to colonize host plants. Moreover, the symptoms developed in host plants infected with the Δ*lov* strain are considerably different from those developed in plants infected with the WT *X. citri* subsp. citri strain, with the presence of necrotic lesions on the infected regions [16]. In order to study potential differences in host responses upon the interaction of *C. sinensis* with the WT and Δ*lov X. citri* subsp. citri strains, we performed a genome-wide transcriptional proﬁling in orange leaves infected with both strains using a *Citrus* cDNA microarray. We found a large number of host genes whose expression profiles were modified upon infection with WT or Δ*lov* strains. Microarray expression for many of the *Citrus* genes was confirmed by real-time RT-PCR analysis. Moreover, various genes were represented by two or more different probes of the microarray, with comparable expression ratios, supporting the reproducibility of the results. A functional categorization of the differentially expressed genes revealed alterations in important biological pathways; the most represented ones being photosynthesis, sucrose and starch catabolism, secondary metabolism, lipid metabolism, cell wall modifications and defense response. This result suggests differences in host responses upon interaction with the different *X. citri* subsp. citri strains. 

During the photosynthesis process, plants convert light energy to chemical energy by generating ATP and reducing power in the form of NADPH. Although the onset of immune reactions and the biosynthesis of defense compounds in infected tissue are energetically demanding, photosynthesis has been shown to be locally down-regulated during plant-pathogen interactions, prioritizing the production of defense-related compounds at the site of infection. Moreover, reductions in the synthesis of primary metabolites can contribute to the depletion of nutrients available for pathogen growth [7]. In our transcriptomic analysis, we observed that during the infection of orange leaves with the Δ*lov* strain of *X. citri* subsp. citri a down-regulation of several photosynthesis-related genes was produced. These genes include components of the LHCs I and II as well as components of the protein complexes of PSI and PSII (Table S1, category Photosynthesis and Figure S3). Accordingly, upon infection of orange leaves with the WT strain of *X. citri* subsp. citri, we could observe a reduction in the photosynthesis rate, indicated by a reduction in the maximum quantum yield of PSII primary photochemistry (Φm_PSII_) and maximum operating efficiency of PSII (OEm_PSII_). Moreover, this effect was much stronger upon infection with the Δ*lov* strain (Figure 2A). Decrease in photosynthesis can be a consequence of the down-regulation of the process or of damage in the photosynthetic apparatus [7-10]. In general, decrease in the parameter Φm_PSII_ is associated with damage of PSII [36] and represents inhibition in the neighboring area of these reaction centers or secondary damage caused by photoinhibition [37]. On the other hand, a decrease in OEm_PSII_ is generally associated with hampered energy transference to open PSII centers, probably as a consequence of alterations in the LHC associated with PSII [38]. These observations suggest that the Δ*lov* infected leaves could present more drastic alterations in the photosynthesis apparatus, mainly in light harvesting and protein components associated with PSII. 

In order to evaluate the possibility of a decrease in photosynthetic pigments, we analyzed the chlorophyll a and b contents in leaves inoculated with *X. citri* subsp. citri WT and Δ*lov* strains. While the amount of both pigments showed a gradual decrease in *X. citri* subsp. citri-inoculated leaves in comparison to the control treatment, no differences were observed between leaves treated with the WT and mutant strains. This indicates that the stronger photosynthesis reduction in *X. citri* subsp. citri Δ*lov*-inoculated leaves is not related to lower chlorophyll content, and, therefore, not associated with differential alterations in the photosynthetic antennae (Figure 2B). This observation suggests the participation of another mechanism for host photosynthesis down-regulation in leaves treated with the mutant strain, such as alterations in the structure of the PSII complexes. This is consistent with the stronger reduction in Φm_PSII_ and OEm_PSII_ parameters in those plants. 

In this context, the Xcc-LOV protein appears to contribute to the maintenance of the photosynthetic efficiency of bacterial-infected tissues. Besides being a way of interfering with plant defense responses, since *X. citri* subsp. citri is a hemibiotrophic plant pathogen which utilizes plant-derived metabolites for growth, maintaining the metabolic integrity of the host plant could be advantageous for the establishment of the bacterium and for disease development. In fact, *X. citri* subsp. citri is known to modulate the photosynthetic apparatus in order to maintain favorable conditions for its own survival [39]. Similar down-regulation of genes involved in photosynthesis has been reported during the *X. citri* subsp. citri-kumquat interaction and is known to favor the maintenance of plant fitness and to direct the plant metabolism to the defense response [40]. Due to the down-regulation of photosynthesis and the simultaneous increased demand for assimilates, changes in carbohydrate metabolism and partitioning have been observed during many plant-pathogen interactions, leading to the conversion of source leaves into sink organs [41,42]. When this happens, sucrose and starch catabolisms are enhanced and the expression and activity of enzymes, such as cell wall invertases and sucrose synthases are induced. Cell wall invertases cleave sucrose in the apoplast into glucose and fructose and the resulting hexoses are transported into the cell. In addition to their role in nutrition, respiration and accumulation of storage compounds, sugars are signals that can regulate gene expression. The hexoses released by the action of invertase activity can act as signaling molecules further repressing photosynthetic genes [43]. Moreover, the cleavage of extracellular sucrose results in the decreased export of assimilates from the tissue, contributing to the starving of apoplast-colonizing pathogens [44]. Increased activities of hexose transporters, the oxidative pentose phosphate pathway, and respiratory metabolism have also been observed upon pathogen interactions, and it is believed that this favors the production of secondary compounds with antimicrobial activity [7]. In our transcriptional analysis, we could observe up-regulation of genes corresponding to enzymes involved in sucrose and starch metabolism, including two invertases and a sucrose synthase upon the inoculation of orange leaves with the Δ*lov* strain of *X. citri* subsp. citri (Figure 3A, Table S1, category CHO Metabolism and Figure S3). The increased expression of these genes, in addition to the more reduced photosynthesis in *X. citri* subsp. citri Δ*lov*-inoculated orange leaves, suggests a metabolic reprogramming of these leaves. Such reprogramming is believed to further enhance the expression of defense-related genes [7]. In fact, this effect was also observed during our transcriptomic analysis where defense-related genes resulted up-regulated upon infection with the Δ*lov* strain. These genes include two types of PR proteins: basic chitinases and PR-4 proteins (Figure 3B, Table S1, category Defense response and Figure S3). These proteins play important roles in plant defense responses upon infection by different types of pathogens [6,45-47].

Several secondary metabolism pathways are known to be the direction of the carbon flow during the primary metabolism reduction in pathogen-infected tissues [7]. During our analysis of the orange transcriptome upon infection with the two *X. citri* subsp. citri strains, we could observe a high representation of secondary metabolism-related genes being differentially regulated. Main differences were observed regarding isoprenoid and phenylpropanoid biosynthesis pathways (Table S1, category Secondary Metabolism). These type of compounds include pigments, membrane sterol lipids, UV-light protectants, phytohormones and antimicrobial phytoalexins [26]. Plant isoprenoids have essential roles in membrane fluidity, respiration, photosynthesis, and regulation of growth and development, and they have specialized functions in plant-pathogen interactions. Phenylpropanoids constitute the monomers of lignin, a polymer that is extremely resistant to microbial degradation. An increase in lignification of plant secondary walls is often observed in response to pathogen attack as an attempt to stop pathogen spreading. In plants, the enzyme PAL catalyses the first step of the phenylpropanoid secondary metabolism. PAL genes are often stimulated by exposure to microbial pathogens and this increase is associated with the lignification of the secondary cell walls [29,30]. In our transcriptomic assay, we detected a rise on the expression of this enzyme, together with other enzymes which operate downstream during lignin biosynthesis, in leaves inoculated with the Δ*lov* strain of *X. citri* subsp. citri compared to those inoculated with the WT strain (Table S1, category Secondary Metabolism and Figure S3). We corroborated this transcriptomic induction by the observation of an increased lignin content associated to fiber-secondary cell walls in Δ*lov*-inoculated leaves compared to the WT and control-treated leaves (Figure 4). This higher cell wall lignification in tissues infected with the mutant strain is indicative of a more efficient plant protective mechanism in order to avoid bacterial spreading.

Additionally, we detected the up-regulation of several genes encoding lipid metabolism proteins upon infection of orange leaves with the Δ*lov* strain of *X. citri* subsp. citri compared to the effect upon WT and control treatments (Table S1, category Lipid Metabolism and Figure S3). Several of these genes have been shown to be involved in plant immunity, such as α/β-hydrolases superfamily proteins, phospholipase D β and phospholipase C-like phosphodiesterases superfamily proteins [31,33]. The fact that those proteins resulted up-regulated in orange leaves treated with the Δ*lov* strain of *X. citri* subsp. citri could be indicative of an increased immunity in those plants. Induction of immunity-related proteins by the mutant strain could indicate that this bacterial mutation results in less production of bacterial effectors that suppress plant immunity. Moreover, alterations in plant cell membranes are involved in signaling pathways related to pathogen attack [48]. Phospholipases hydrolyze phospholipids, which are the backbones of biological membranes. The generation of second messengers by degradation of membrane lipids is an essential feature of some signal transduction pathways activated in response to hormones, senescence and environmental stress such as pathogen attack [49]. The up-regulation of phospholipase D (Figure 5A and Figure S3) in orange leaves inoculated with the mutant *X. citri* subsp. citri strain could be indicative of the activation of a signaling cascade related to plant defense responses.

We also evaluated plant cell membranes integrity and the results indicated a more drastic integrity loss in orange leaves inoculated with the Δ*lov* strain of *X. citri* subsp. citri compared to those inoculated with the WT strain, which can be the consequence of higher tissue damage at the site of infection (Figure 5B). In fact, the organization and structure of tissues inoculated with the Δ*lov X. citri* subsp. citri strain showed serious alterations, with extensive damage and mesophyll disorganization (Figure 5C). Consistent with these findings, we have previously shown that the Δ*lov* strain produced phenotypically different lesions in orange leaves than those rendered by the WT, generating visible necrotic regions on the infected tissues [16]. Similar patterns of tissue damage have been previously observed upon *Citrus* non-host responses to the bacterium *Xanthomonas campestris* pv. *vesicatoria* [6]. Tissue disorganization and damage have also been observed in kumquat upon *X. citri* subsp. citri infection [40]. 

Finally, we found a strong up-regulation of a *Citrus* EST corresponding to an endo-β-mannanase upon orange inoculation with the Δ*lov* strain of *X. citri* subsp. citri (Figure 5A and Table S1, category Cell Wall). This enzyme hydrolyzes the β-1,4-linkages in the backbone of mannans (one of the plant cell wall hemicelluloses) and plays important roles in plant growth and developmental events in which cell wall degradation is involved [50]. This observation provides further evidence of plant cell wall structure alterations in tissues inoculated with the Δ*lov X. citri* subsp. citri strain. 

The increased expression of enzymes involved in sucrose catabolism and secondary metabolism, the induction of lipid metabolism enzymes implicated in plant immunity, the enhanced biochemical and structural host tissue alterations such as lignin deposition and tissue disorganization, as well as the stronger photosynthesis reduction observed in tissues inoculated with the Δ*lov* strain of *X. citri* subsp. citri suggest a stronger host response upon infection with this strain compared to the effect observed with the WT strain. Thus, as postulated in our previous work, the Xcc-LOV protein would participate in the regulation of the virulence process [16]. Several of these effects resemble responses observed during the orange non-host interaction with *X. campestris* pv. vesicatoria, where modifications in the expression profiles of genes involved in photosynthesis, defense and biosynthesis of secondary metabolites including lignin have been observed. Increased membrane permeability and host tissue disorganization were also observed in orange leaves inoculated with *X. campestris* pv. vesicatoria compared to the effects observed upon infection with *X. citri* subsp. citri, but on a shorter timescale [6]. Moreover, differences in the PAL gene induction have also been observed between compatible and incompatible interactions, taking place earlier or in a more pronounced manner in the incompatible ones [51,52]. Additionally, induction of phospholipase D has been reported for several plant-pathogen interactions, including that of *Xanthomonas oryzae* pv. oryzae and rice, where accumulation of this enzyme at the plasma membranes in the site of infection was observed only during incompatible interactions [32,33,53]. Finally, it has been shown that induction of PR proteins is stronger during incompatible interactions in comparison to compatible interactions for many plant species including *C. sinensis* [6,45-47]. Considering these observations, the interaction of the Δ*lov* strain of *X. citri* subsp. citri with orange leaves, despite being a compatible interaction, resembles many aspects of incompatible interactions. Therefore, we hypothesize the existence of “more efficient defense response” in orange plants infected with the Δ*lov* strain. On the other hand, *in planta* bacterial growth was not significantly affected in this strain [16], which suggests that this increased plant response is insufficient to avoid pathogen multiplication. This effect is probably due to a plethora of bacterial mechanisms and effectors that are still active in the Δ*lov* mutant strain and that enable bacterial propagation. In this context, the Xcc-LOV protein from *X. citri* subsp. citri and environmental light absorption through this protein would be involved in a bacterial mechanism aimed at reducing part of the plant defense response, thus allowing the maintenance of host metabolism and tissue integrity, which is of crucial importance for a hemibiotrophic pathogen. As the Xcc-LOV protein is a sensing protein, acting at the early steps of a signal transduction system, this mechanism probably involves the downstream activation of bacterial components involved in the pathogenicity process. Moreover, as *X. citri* subsp. citri genome presents three additional genes coding for putative photoreceptors [two genes encoding BLUF (Blue-light sensing using flavin) proteins and one encoding a phytochrome], it is probable that more than one photoreceptor act in concert to activate this bacterial mechanism. 

The results presented here show the novel participation of a light-sensing bacterial protein in the counteraction of plant defense responses and represent the potential pathogen employment of light, an environmental factor important for plant defense response, in order to successfully infect its host.

## Materials and Methods

### Bacterial strains and growth conditions

*Xantomonas citri* subsp. citri (Hasse) strains were derivatives of strain 99-1330, which was kindly provided by Blanca I. Canteros (INTA Bella Vista, Argentina). *X. citri* subsp. citri *lov* mutant (Δ*lov*) strain construction was described by Kraiselburd et al [16]. Bacteria were grown aerobically at 28 °C with shaking at 200 rpm in Silva Buddenhagen (SB) medium [34] supplemented with 25 μg/mL ampicillin (Amp) for the WT strain, and 50 µg/mL streptomycin (Sm) for the Δ*lov* strain.

### Plant material and inoculation

*Citrus sinensis* cv. Valencia late orange plants were kindly provided by Catalina Anderson and Gastón Alanis (INTA Concordia, Argentina). Plants were grown in a greenhouse with a photoperiod of 14 h light (150 μE.m^-2^.s^-1^) and 10 h dark at a temperature of 25 °C and 80 % humidity. For plant inoculation, bacteria were cultured in SB broth to an optical density at 600 nm (OD_600_) of 1 and cultures were adjusted to 10^7^ colony forming units (CFU)/mL with 10 mM MgCl_2_. Bacterial suspensions were infiltrated into the abaxial leaf surface using a needleless syringe. 10 mM MgCl_2_ was used as a negative control for non-infected leaves. 

### Plant mRNA purification and labeling

Orange leaves were inoculated with WT and Δ*lov* strains of *X. citri* subsp. citri and with 10 mM MgCl_2_. For each treatment, three leaves were completely infiltrated, each belonging to an independent plant. Inoculated leaves were taken at 24 h after infiltration treatments for total RNA extraction using Trizol^®^ Reagent (Invitrogen). For each treatment, RNA was extracted from three biological replicates and independently processed, labeled and hybridized to different microarrays.

The quality and quantity of the mRNA samples were verified by agarose gel electrophoresis and UV spectroscopy [54]. Then, 5 µg of total RNA were reversely transcribed with 0.5 µL T7 Oligo/(dT) primer and 0.5 µL ArrayScript™ reverse transcriptase (Ambion) in the presence of 0.5 mL of RNase inhibitor (Ambion). Second strand cDNA synthesis was performed with 1 µL of *Escherichia coli* DNA polymerase I and 0.5 µL RNase H (Ambion). *In vitro* transcription to synthesize Amino Allyl-Modified RNA (aRNA) from the double-stranded cDNA templates and aRNA purification were performed using the Amino Allyl MessageAmp™ II aRNA Amplification Kit following the manufacturer’s instructions. 

For fluorescent labeling, 5 µg of aRNA were vacuum dried, resuspended in 4 µL sodium carbonate buffer (0.1 M Na_2_CO_3_ in DEPC-treated water, pH 8.5) and mixed with 4 µL of a 10 mM solution of either Cy3^TM^ or Cy5^TM^ (Amersham) in dimethyl sulfoxide. Samples were incubated at room temperature in the dark for 1 h and labeled aRNA was purified using the RNeasy Plant Minikit (Qiagen). Dye incorporation was determined spectrophotometrically.

### Microarray hybridization and data analysis

For the microarray assay we used *Citrus* cDNA microarray slides consisting of 21.081 cDNA probes, generated by the Spanish Citrus Functional Genomics Project (CFGP) [55]. These probes correspond to *Citrus*-expressed sequence tags (EST) from different gene libraries [56]. For microarray hybridization, Cy5-labelled aRNA synthesized from each individual mRNA sample and Cy3-labelled aRNA synthesized from a reference sample consisting of a mixture of equal amounts of RNA from all experimental samples were combined in equal amounts (200 pmoles of each dye) and fragmented using the RNA Fragmentation Reagents (Ambion). Fragmented samples were incubated for 2 min at 80 °C, mixed with 50 µL of pre-heated hybridization buffer [5X saline-sodium citrate (SSC), 50 % formamide, 0.1 % sodium dodecyl sulfate (SDS), 0.1 mg/mL salmon sperm DNA] and applied to the microarray slide prehybridized in 5X·SSC, 0.1 % SDS, 1 % bovine serum albumin (BSA). Hybridization was performed overnight at 42 °C. After hybridization, slides were washed twice with 2X·SSC, 0.1 % SDS for 5 min at 42 °C, followed by two washes with 0.1X·SSC, 0.1 % SDS for 5 min at room temperature, and 5 washes with 0.1X SSC for 1 min at room temperature. Finally, slides were briefly rinsed in 0.01X·SSC before being dried by centrifugation at 300 rpm during 3 min. 

Hybridized arrays were scanned with a Scanarray Gx scanner (PerkinElmer) at wavelengths of 543 nm and 633 nm for the fluorophores Cy3 and Cy5, respectively, using the Scanarray Express software to obtain an appropriate photomultiplier gain ratio for the two channels. The GenePix 4.1 software (Axon Instruments) was used to transform the intensity into numeric data. The raw microarray data of the 9 hybridizations and the protocols used to produce the data were deposited in the ArrayExpress database under the accession number E-MEXP-3975. The normalization and analysis of microarrays were carried out using the package “Linear Models in Microarrays” (LIMMA) of the "R" software (bioconductor project, www.bioconductor.org). Normalization and background elimination was carried out using the Lowess method. Data were selected as significant when having p-values corrected by false discovery rate correction (FDR) lower than 0.01 and M values cutoff of ±1, being M=log_2_[*X. citri* subsp. citri Δlov /control], M=log_2_[*X. citri* subsp. citri WT/Control] and M=log_2_[*X. citri* subsp. citri WT/X*. citri* subsp. citri Δ*lov*] for *X. citri* subsp. citri Δ*lov*-control, *X. citri* subsp. citri WT-control and *X. citri* subsp. citri Δlov-*X. citri* subsp. citri WT comparisons, respectively. Venn diagrams were constructed using the Venny tool (http://bioinfogp.cnb.csic.es/tools/venny/index.html). To assign a possible function to the differentially expressed ESTs, the corresponding sequences (obtained from the database CFGP, http://citrusgenomics.ibmcp-ivia.upv.es) and the complete sequence of the corresponding orange genes (obtained from the *C. sinensis* phytozome database at http://www.phytozome.net/citrus.php) were compared with the National Center for Biotechnology Information (NCBI, www.ncbi.nlm.nih.gov) and The *Arabidopsis* Information Resource (TAIR, www.arabidopsis.org) data bases using the BLASTX algorithm. The *Arabidopsis* Homologues with an expected value (E-value) <10 E^-03^ were grouped into functional categories using the agriGO web-based platform in order to identify the over-represented biological processes [24]. The results were also correlated with the classification made by the MapMan software [25].

### Real-time RT-PCR

Gene expression was confirmed through real-time RT-PCR analysis. Primers were designed using Primer3 v.0.4.0 software [57]. The analysed ESTs, primer sequences and product lengths are indicated in Table S2. cDNA was synthesized from 1 μg of total RNA from the same samples used in the microarray experiments through M-MuLV Retro Transcriptase enzyme (Promega, USA) and d(T)15 oligonucleotide following the manufacturer’s instructions. PCR products using genomic DNA or cDNA templates for the housekeeping gene (actin) were sized differently, allowing for the detection of genomic DNA contamination. Real-time PCR was performed with an Applied Biosystems instrument equipped with Stepone Software version 2.3. Reactions were performed with 5 μL of 1/20 dilutions of cDNA template and a homemade SYBR green-I reaction mixture [58] containing 1:50000 diluted SYBR green-I (Invitrogen), 10 pmol of each primer, 0.5 U Platinum-Taq DNA polymerase (Invitrogen), 40 mmol dNTPs, 3.75 mM MgCl_2_ and 1X Platinum-Taq buffer in a final volume of 20 μL under the following conditions: 95 °C for 1 min followed by 40 cycles of 95 °C for 15 s, 58 °C for 30 s and 72 °C for 45 s. Fluorescent intensity data were acquired during the 72 °C extension step. The specificity of the amplification reactions was assessed by melting curves analysis, which were run at 95 °C for 15 s and 60 °C for 15 s followed by an increase in temperature from 60 to 85 °C (0.2 °C/s) with continuous fluorescence recording. To perform the analysis of relative expression we used the 2^-∆∆CT^ method [59] normalizing to actin.

### Determination of photosynthetic parameters

Chlorophyll fluorescence measurements were performed using a pulse-modulated fluorometer (Qubit Systems, ttp://qubitsystems.com) and a MINI-PAM 2000 fluorometer (Walz, http://www.walz.com). Orange leaves were inoculated with WT and Δ*lov* strains of *X. citri* subsp. citri and with 10 mM MgCl_2_. Three independents biological samples were used. At different times after inoculation, leaves were incubated in the darkness for 30 min and then exposed to a constant actinic light (200 mmol m^-2^ sec^-1^), when the value F_0_ (minimum chlorophyll fluorescence in dark adapted leaves) was determined. Next, light induction measurements were performed on these dark-adapted leaves by application of a saturating pulse to obtain the F_m_ value (maximum chlorophyll fluorescence in dark adapted leaves). The F_v_ value (variable fluorescence) corresponds to F_m_-F_0_. Saturating pulses were applied every 30 sec to obtain the fluorescence in light adapted leaves (F’) and the corresponding F_v_’ and F_m_’ values after each light period. The photosynthetic parameters maximum quantum yield of PSII (Φm_PSII_= F_v_/F_m_) and maximum operating efficiency of PSII (OEm_PSII_= F’_v_ /F’_m_) were calculated as described by Baker [36]. 

### Pigment determination

For chlorophylls quantification orange leaves were inoculated with suspensions of the WT and Δ*lov* strains of *X. citri* subsp. citri and with 10 mM MgCl_2_. Three independents biological samples were used. Two leaf discs of 0.8 cm-diameter were taken for each treatment at different times after inoculation and placed in 1.5 mL tubes containing 1 mL of N,N-dimethylformamide. Samples were incubated for 72 h at room temperature in darkness. After incubation the absorbance of the samples at 664 and 647 nm was recorded. Total chlorophyll, chlorophyll a and chlorophyll b contents were determined according to the equations described by Porra et al. [60]. 

### Histological analysis

Orange leaves were inoculated with suspensions of the WT and Δ*lov* strains of *X. citri* subsp. citri and with 10 mM MgCl_2_. Three independents biological samples were used. At different times after inoculation leaves were removed from the plant and fragments of around 1 cm^2^ were excised from each inoculated region. Leaf fragments were fixed in FAA solution (10 % formaldehyde, 5 % glacial acetic acid, 50 % ethanol) for 24 h. After fixation, samples were dehydrated and embedded in paraffin. Cross-sections were cut using a Minot type microtome, deparaffinized and stained with safranin-fast Green to be observed with a PM-10ADS Olympus Automatic Photomicrographic Systemlight microscopy [61]. Alternative, leaf fragments were embedded and frozen in O.C.T (optimal cutting temperature) compound. Frozen tissues were sliced using a Microm Zeiss HM 500 cryostat at -20 °C and the 10 µm sections were mounted on a glass slide and stained for lignin determination. Lignin staining was performed by covering the samples with acidified fluoroglucin (1 % in ethanol, acidified with a drop of HCl) [62] followed by observation with a PM-10ADS Olympus Automatic Photomicrographic Systemlight microscopy.

### Ion leakage assay

For ion leakage measurements orange leaves were inoculated with suspensions of the WT and Δ*lov* strains of *X. citri* subsp. citri as previously described, but distilled water was used for bacterial OD_600_ adjustment and for the control treatment. Three independent biological samples were used. At different times after inoculation 0.8 cm-diameter leaf discs were removed and floated for 15 min in a 12-well polystyrene plate containing 2 mL of distilled water. The discs were transferred to 1.5 tubes containing 1 mL distilled water and the conductance was measured after 24 h of incubation at room temperature. The conductance of boiled leaf discs was taken as 100 % ion content [34]. The percentage of ion leakage was calculated according to the following equation:

Percentage of ion leakage = (conductivity before boiling/ conductivity after boiling)*100

### Statistical analysis

Quantitative analyses were performed with at least three independent biological samples. Data were subjected to a multifactorial ANOVA and Tukey’s multiple comparison tests using Infostat software (Infostat 2006H, http/ www.infostat.com.ar).

## Supporting Information

Figure S1 **Illustration of over-represented families of differentially expressed genes during *Citrus sinensis* interaction with *Xanthomonas citri* subsp. citri WT and Δ*lov* strains, for genes with M** **> 1, where M is log_2_[expression for WT treatment/expression for Δ*lov* treatment]**. The Gene Ontology category (GO term) and p-value with false discovery rate correction (FDR) are indicated together with the relations of number of genes with modified expression during the response/number of genes in the input list and of number of genes represented in the microarray/total number of genes in the reference list. Colors of squares indicate significance levels (growing significance level from yellow to red) and arrows indicate the relationship between categories: “is a” (black line), “negative regulates” (green line), “one significant node” (thin dotted line) and “two significant nodes” (thick dotted line).
(TIF)

Figure S2 **Illustration of over-represented families of differentially expressed genes during *Citrus sinensis* interaction with *Xanthomonas citri* subsp. citri WT and Δ*lov* strains, for genes with M** **< -1, where M is log_2_[expression for WT treatment/expression for Δ*lov* treatment]**. The Gene Ontology category (GO term) and p-value with false discovery rate correction (FDR) are indicated together with the relations of number of genes with modified expression during the response/number of genes in the input list and of number of genes represented in the microarray/total number of genes in the reference list. Colors of squares indicate significance levels (growing significance level from yellow to red) and arrows indicate the relationship between categories: “is a” (black line), “negative regulates” (green line), “one significant node” (thin dotted line) and “two significant nodes” (thick dotted line).
(TIF)

Figure S3 **Real-time RT-PCR (qRT-PCR) analysis for the validation of microarray expression of *Citrus sinensis* genes during the interaction of orange leaves with *Xanthomonas citri* subsp. citri WT and Δ*lov* strains, and during the control treatment (10 mM MgCl_2_, Ctrl).** The log_2_ of the expression ratios between treatments values (M) are shown for genes belonging to the functional categories photosynthesis (Phot 1: photosystem II reaction center protein, Phot 2: plastocyanin 1), CHO metabolism (CHO 1: cell wall invertase), secondary metabolism (Pal 1: phenylalanine ammonia lyase 1), lipid metabolism (Lip 1: fatty acid desaturase 2, Lip 2: phospholipase D β 1), and biotic stress (def 1: basic chitinase, def 2: pathogenesis-related 4 protein) are presented. Data are averages of values obtained from three independent biological samples. Bars represent standard error. Primer and product information is available in Table S2.
(TIF)

Figure S4 **Complementation assays with the Δ*lov*-p*lov* strain of *Xanthomonas citri* subsp. citri.** (A) Expression analysis of *Citrus sinensis* genes upon orange interaction with the WT, Δ*lov* and Δ*lov*-p*lov* strains of *Xanthomonas citri* subsp. citri by real-time RT-PCR. The log_2_ of the expression ratio between treatments (M) are shown for the genes Phot 1: photosystem II reaction center protein, Phot 2: plastocyanin 1, CHO 1: cell wall invertase, Pal 1: phenylalanine ammonia lyase 1 and Lip 1: fatty acid desaturase 2. Data are averages of values obtained from three independent biological samples. Bars represent standard error. Primer and product information is available in Table S2. (B) Histological analysis of lignin deposition in orange leaves upon interaction with *X. citri* subsp. citri by acid fluoroglucin staining. Stained tissues were observed using a visible microscope with a 1000x magnification at 7 days after leaf inoculation with the WT, Δ*lov* and Δ*lov-*p*lov* strains of this bacterium. The wine-red coloration represents lignin deposition in plant secondary cell walls. Scale bars: 5 µm. Identical results were obtained with three independent biological samples. (C) Ion leakage measurements of orange leaves at 5 and 8 days post inoculation (dpi) with the WT, Δ*lov* and Δ*lov-*p*lov* strains of *X. citri* subsp. citri and with water (control). Results are expressed as percentage of ion leakage and correspond to the mean of three independent biological replicates. Error bars represent standard errors and asterisks indicate significant differences between Δ*lov* and WT or Δ*lov*-p*lov* treatments (p<0.05). (D) Tissue integrity of orange leaves inoculated with WT, Δ*lov* and Δ*lov-*p*lov* *X. citri* subsp. citri strains. Tissues were analyzed by safranine/fast green staining. Panels show the microscopic visualization of stained tissue fragments 7 days after bacterial and control treatments using a 400x magnification. Scale bars: 10 µm.
(TIF)

Methods and Results S1 **Complementation assays.**
(DOC)

Table S1 **Differentially regulated *Citrus sinensis* ESTs during the interaction with *Xanthomonas citri* subsp. citri WT and Δ*lov* strains related to the functional categories photosynthesis, CHO metabolism, secondary metabolism, lipid metabolism, cell wall modification and biotic stress.** The corresponding *C. sinensis* complete genes (http://www.phytozome.net/citrus.php) are specified. Homologies to sequences in *Arabidopsis thaliana* non-redundant databases are indicated. Empty rows indicate identity to previous proteins. The log_2_ of expression ratio between treatments (M), together with false discovery rate correction (FDR) and standard error (SE) are shown. Data are averages of three repetitions corresponding to three independent biological samples.
(XLS)

Table S2 ***Citrus sinensis* ESTs analyzed for the validation of microarray data by real-time RT-PCR.** ID, primer abbreviation, *C. sinensis* complete gene (http://www.phytozome.net/citrus.php), primer sequences and length of the amplified fragment are indicated for each EST. Primer design was performed with the Primer3 v.0.4.0 software [57].
(DOC)
