# Supplementary figures and images for: The LOV Protein of Xanthomonas citri subsp. citri Plays a Significant Role in the Counteraction of Plant Immune Responses during Citrus Canker
Source: PLoS One. 2013 Nov 15;8(11):e80930. doi: 10.1371/journal.pone.0080930 (PMC3829917; doi:10.1371/journal.pone.0080930)

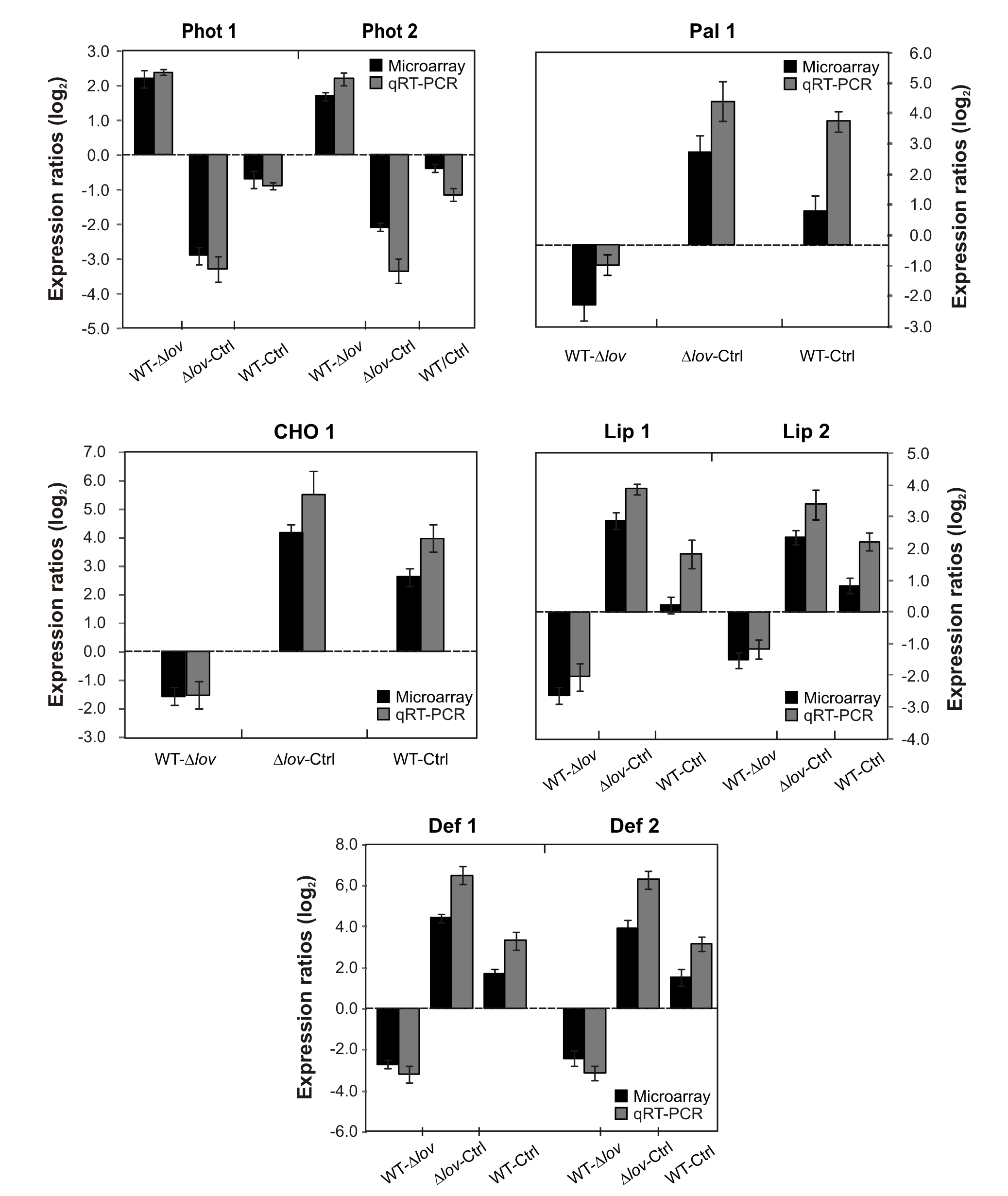

Supplement: Figure S3 — Real-time RT-PCR (qRT-PCR) analysis for the validation of microarray expression of Citrus sinensis genes during the interaction of orange leaves with Xanthomonas citri subsp. citri WT and Δlov strains, and during the control treatment (10 mM MgCl2, Ctrl). The log2 of the expression ratios between treatments values (M) are shown for genes belonging to the functional categories photosynthesis (Phot 1: photosystem II reaction center protein, Phot 2: plastocyanin 1), CHO metabolism (CHO 1: cell wall invertase), secondary metabolism (Pal 1: phenylalanine ammonia lyase 1), lipid metabolism (Lip 1: fatty acid desaturase 2, Lip 2: phospholipase D β 1), and biotic stress (def 1: basic chitinase, def 2: pathogenesis-related 4 protein) are presented. Data are averages of values obtained from three independent biological samples. Bars represent standard error. Primer and product information is available in Table S2. (TIF) [file pone.0080930.s003.tif]

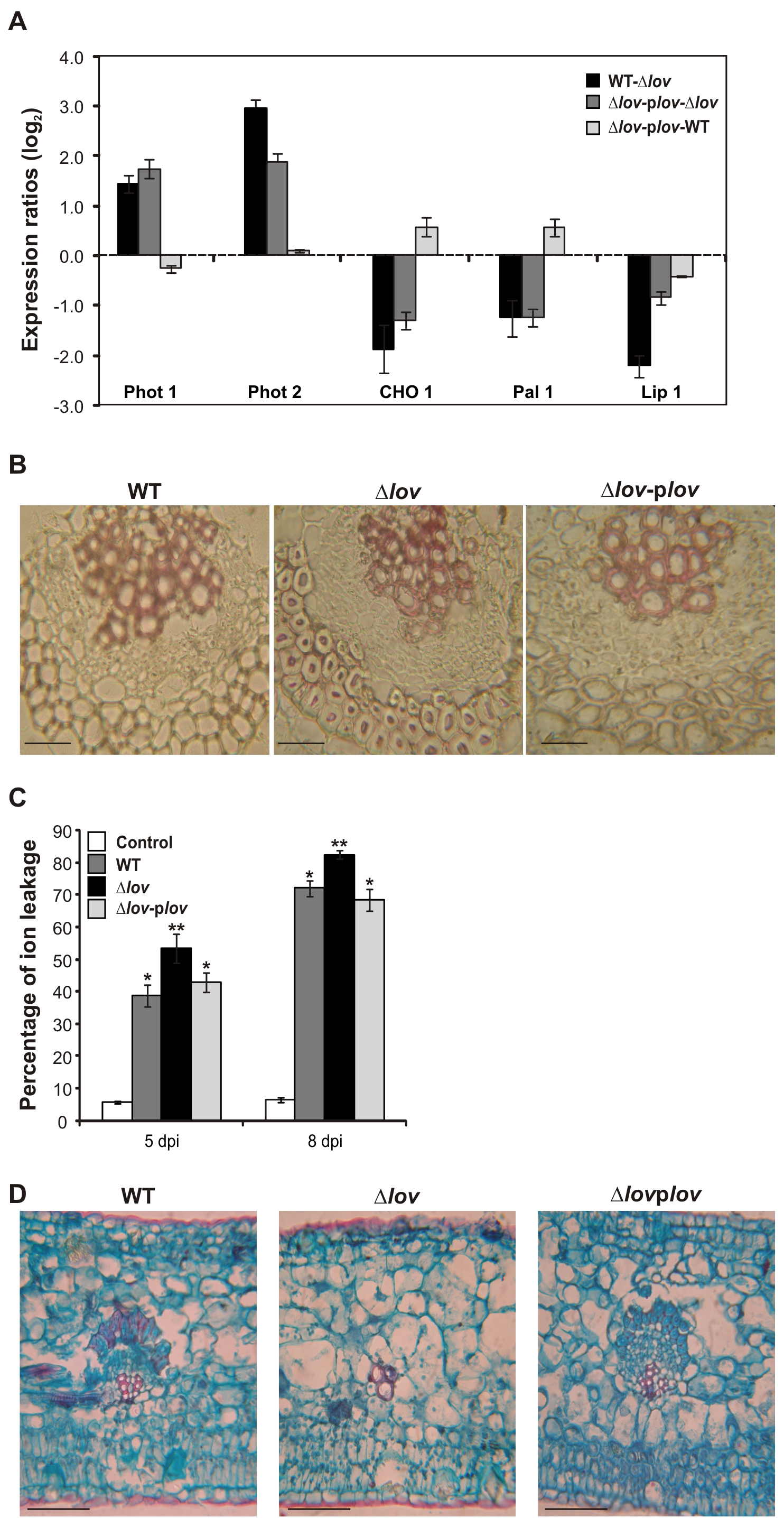

Supplement: Figure S4 — Complementation assays with the Δlov-plov strain of Xanthomonas citri subsp. citri. (A) Expression analysis of Citrus sinensis genes upon orange interaction with the WT, Δlov and Δlov-plov strains of Xanthomonas citri subsp. citri by real-time RT-PCR. The log2 of the expression ratio between treatments (M) are shown for the genes Phot 1: photosystem II reaction center protein, Phot 2: plastocyanin 1, CHO 1: cell wall invertase, Pal 1: phenylalanine ammonia lyase 1 and Lip 1: fatty acid desaturase 2. Data are averages of values obtained from three independent biological samples. Bars represent standard error. Primer and product information is available in Table S2. (B) Histological analysis of lignin deposition in orange leaves upon interaction with X. citri subsp. citri by acid fluoroglucin staining. Stained tissues were observed using a visible microscope with a 1000x magnification at 7 days after leaf inoculation with the WT, Δlov and Δlov-plov strains of this bacterium. The wine-red coloration represents lignin deposition in plant secondary cell walls. Scale bars: 5 µm. Identical results were obtained with three independent biological samples. (C) Ion leakage measurements of orange leaves at 5 and 8 days post inoculation (dpi) with the WT, Δlov and Δlov-plov strains of X. citri subsp. citri and with water (control). Results are expressed as percentage of ion leakage and correspond to the mean of three independent biological replicates. Error bars represent standard errors and asterisks indicate significant differences between Δlov and WT or Δlov-plov treatments (p<0.05). (D) Tissue integrity of orange leaves inoculated with WT, Δlov and Δlov-plov X. citri subsp. citri strains. Tissues were analyzed by safranine/fast green staining. Panels show the microscopic visualization of stained tissue fragments 7 days after bacterial and control treatments using a 400x magnification. Scale bars: 10 µm. (TIF) [file pone.0080930.s004.tif]
