# Supplementary material for: The LOV Protein of Xanthomonas citri subsp. citri Plays a Significant Role in the Counteraction of Plant Immune Responses during Citrus Canker
Source: PLoS One. 2013 Nov 15;8(11):e80930. doi: 10.1371/journal.pone.0080930 (PMC3829917; doi:10.1371/journal.pone.0080930)
Supplement: Methods and Results S1 — Complementation assays. (DOC) [file pone.0080930.s005.doc]

**The LOV protein of *Xanthomonas citri* subsp. citri plays a significant role in the counteraction of plant immune responses during citrus canker**

**Ivana Kraiselburd^1^, Lucas D. Daurelio^1^, María Laura Tondo^1^, Paz Merelo^2^, Adriana A. Cortadi^3^, Manuel Talón^2^, Francisco R. Tadeo^2^, Elena G. Orellano^1^,***

**Supporting Methods and Results: Complementation assays**

Construction of the *Xanthomonas citri* subsp. citri Δ*lov* strain complemented with a wild type *lov* gene (Δ*lov-*p*lov*) was described by Kraiselburd *et al* [1]. This bacterium was grown aerobically at 28 ºC with shaking at 200 rpm in Silva Buddenhagen (SB) medium [2], supplemented with 50 µg/mL streptomycin (Sm) and 40 µg/mL gentamycin (Gm).

For plant inoculation, *X. citri* subsp. citri WT, Δ*lov* and Δ*lov-*p*lov* strains were cultured in SB broth to an optical density at 600 nm (OD_600_) of 1 and cultures were adjusted to 10^7^ colony forming units (CFU)/mL with 10 mM MgCl_2_. Bacterial suspensions were infiltrated into the abaxial surface of *Citrus sinensis* cv. Valencia late orange leaves using a needleless syringe. 10 mM MgCl_2_ was used as a negative control for non-infected leaves. Plants were grown in a greenhouse with a photoperiod of 14 h light (150 μE.m^-2^.s^-1^) and 10 h dark at a temperature of 25 °C and 80 % humidity.

In order to perform gene expression analysis, three orange leaves were completely infiltrated for each treatment, each leaf belonging to an independent plant. Inoculated leaves were taken at 24 h after infiltration for total RNA extraction using Trizol® Reagent (Invitrogen). For each treatment, RNA was extracted from three biological replicates and independently processed. cDNA was synthesized from 1 μg of total RNA samples through M-MuLV Retro Transcriptase enzyme (Promega, USA) and d(T)15 oligonucleotide following the manufacturer’s instructions. Gene expression was analysed through real-time RT-PCR analysis using a subset of the primers described in Table S2. Real-time RT PCR was performed with an Applied Biosystems instrument equipped with Stepone Software version 2.3. Reactions were performed with 5 μL of 1/20 dilutions of cDNA template and a homemade SYBR green-I reaction mixture [3] containing 1:50000 diluted SYBR green-I (Invitrogen), 10 pmol of each primer, 0.5 U Platinum-Taq DNA polymerase (Invitrogen), 40 mmol dNTPs, 3.75 mM MgCl2 and 1X Platinum-Taq buffer in a final volume of 20 μL under the following conditions: 95 °C for 1 min followed by 40 cycles of 95 °C for 15 s, 58 °C for 30 s and 72 °C for 45 s. Fluorescent intensity data were acquired during the 72 ºC extension step. The specificity of the amplification reactions was assessed by melting curve analysis, which were run at 95 ºC for 15 s and 60 ºC for 15 s followed by an increase in temperature from 60 to 85 ºC (0.2 ºC/s) with continuous fluorescence recording. To perform the analysis of relative expression we used the 2^-∆∆CT^ method [4] normalizing to actin (housekeeping gene).

**Figure S4A** shows the log_2_ of the expression ratios between treatments values for genes belonging to the functional categories photosynthesis, CHO metabolism, secondary metabolism, and lipid metabolism. We could observe that the expression of the assayed *Citrus* genes presented similar behaviour upon treatment with the WT and complementant strain.

In order to evaluate lignin deposition in infected tissues, orange leaves were inoculated with 10^7^ CFU/mL suspensions of the WT, Δ*lov* and Δ*lov*-p*lov* strains of *X. citri* subsp. citri. Three independents biological samples were used. At 7 days after inoculation leaves were removed from the plant and fragments of around 1 cm^2^ were excised from the inoculated region. Leaf fragments were embedded and frozen in O.C.T (optimal cutting temperature) compound. Frozen tissues were sliced using a Microm Zeiss HM 500 cryostat at -20 ºC and the 10 µm sections were mounted on a glass slide. Lignin staining was performed by covering the samples with acidified fluoroglucin (1 % in ethanol, acidified with a drop of HCl) [5] followed by observation with a PM-10ADS Olympus Automatic Photomicrographic Systemlight microscopy.

Infected tissues showed a characteristic lignin staining around the xylem vessels and on the sclerenchymatous fibers surrounding the vascular bundles. However, leaves inoculated with the Δ*lov* strain showed an increased lignin content in the cellular lumen of sclerenchymatous fibers compared to tissues inoculated with the WT and Δ*lov*-p*lov* strains, indicated by more intense wine-red coloration **(Figure S4B)**. These results show the reversion of the WT phenotype in leaves inoculated with the Δ*lov-plov* of *X. citri* subsp. citri.

For ion leakage measurements orange leaves were inoculated with suspensions of the WT, Δ*lov* and Δ*lov*-p*lov* strains of *X. citri* subsp. citri as described previously, but bacterial cultures were adjusted to 10^7^ CFU/mL with distilled water. Control treatment was also performed with distilled water. Three independents biological samples were used. At different times after inoculation 0.8 cm-diameter leaf discs were removed and floated for 15 min in a 12-well polystyrene plate containing 2 mL of distilled water. The discs were transferred to 1.5 tubes containing 1 mL distilled water and the conductance was measured after 24 h of incubation at room temperature. The conductance of boiled leaf discs was taken as 100 % ion content [2]. The percentage of ion leakage was calculated as follows:

Percentage of ion leakage = (conductivity before boiling/ conductivity after boiling)*100

When ion leakage assay was measured five days after treatment, increased tissue permeability was observed for bacterial-inoculated orange leaves compared to the control treatment. However, this increase resulted more intense in the Δ*lov*-treated orange leaves, compared to the WT and Δ*lov*-p*lov*-treated ones **(Figure S4C)**. This result indicates a more drastic effect in plant tissue integrity upon infection with the mutant strain compared to the WT and Δ*lov*-p*lov* strains and verifies the complementation ability of the complementant strain.

In order to evaluate tissue integrity and structure, orange leaves were inoculated with suspensions of the WT, Δ*lov* and Δ*lov*-p*lov* strains of *X. citri* subsp. citri and with 10 mM MgCl_2_. Three independents biological samples were used. At 7 days after inoculation leaves were removed from the plant and fragments of around 1 cm^2^ were excised from the inoculated region. Leaf fragments were fixed in FAA solution (10 % formaldehyde, 5 % glacial acetic acid, 50 % ethanol) for 24 h. After fixation, samples were dehydrated and embedded in paraffin. Cross-sections were cut using a Minot type microtome, deparaffinized and stained with safranin-fast Green to be observed with a PM-10ADS Olympus Automatic Photomicrographic Systemlight microscopy [6].

Tissue structure showed a low level of damage after infection with the WT and Δ*lov*-p*lov* strain of *X. citri* subsp. citri. However, severe structure disorganization and mesophyll cell lysis were observed in *X. citri* subsp. citri Δ*lov*-treated tissues **(Figure S4D)**.

**REFERENCES**

1. Kraiselburd I, Alet AI, Tondo ML, Petrocelli S, Daurelio LD, et al. (2012) A LOV protein modulates the physiological attributes of Xanthomonas axonopodis pv. citri relevant for host plant colonization. PLoSOne 7: e38226.

2. Daurelio LD, Tondo ML, Dunger G, Gottig N, Ottado J, et al. (2009) Hypersensitive response. In: Sampietro DA, Vattuone MA, atal n CAN, olyticka B, editors. Plant Bioassays Huston, Texas. pp. 187-206.

3. Karsai A, Muller S, Platz S, Hauser MT (2002) Evaluation of a homemade SYBR green I reaction mixture for real-time PCR quantification of gene expression. Biotechniques 32(4):790-2, 794-6.

4. Livak KJ, Schmittgen TD (2001) Analysis of relative gene expression data using real-time quantitative PCR and the 2 -( DDCT) method. Methods 25(4): 402-8.

5. WHO (World Health Organization) (2011) Quality Control Methods for Herbal Materials. WHO Press, World Health Organization Geneva, Switzerland.

6. D'Ambrogio de Argüeso A (1986) Manual de técnicas en histología vegetal. Buenos Aires: Editorial Hemisferio Sur S.A.
