# Supplementary material for: The LOV Protein of Xanthomonas citri subsp. citri Plays a Significant Role in the Counteraction of Plant Immune Responses during Citrus Canker
Source: PLoS One. 2013 Nov 15;8(11):e80930. doi: 10.1371/journal.pone.0080930 (PMC3829917; doi:10.1371/journal.pone.0080930)
Supplement: Table S2 — Citrus sinensis ESTs analyzed for the validation of microarray data by real-time RT-PCR. ID, primer abbreviation, C. sinensis complete gene (http://www.phytozome.net/citrus.php), primer sequences and length of the amplified fragment are indicated for each EST. Primer design was performed with the Primer3 v.0.4.0 software [57]. (DOC) [file pone.0080930.s007.doc]

| **Primer set** | **Product description** | **ID** | ***Citrus sinensis* complete gene** | **Forward primer 5’-3’ sequence** | **Reverse primer 5’-3’ sequence** | **Product size (bp)** |
| --- | --- | --- | --- | --- | --- | --- |
| **Phot 1** | photosystem II reaction center | C31604G05 | orange1.1g032691m | TTACACGCTCGGTTCTTGTG | AAATGGAAGCCCTGTTCCTT | 251 |
| **Phot 2** | plastocyanin 1 | IC0AAA26DC03 | orange1.1g030950m | TGGCTTTTGTTCCAAGCTCT | CCTTGGTGAGGGGAACAGTA | 232 |
| **CHO 1** | cell wall invertase | C06024D04 | orange1.1g008242m | TTGGCCCTGATAAGAGATGG | AGGACACTCCCACATTCCTG | 152 |
| **Pal 1** | phenylalanine ammonia lyase 1 | C32004A10 | orange1.1g005031m | CTGAAGTCATGCAAGGCAAA | GGTTGCAAACCGAATCACTT | 242 |
| **Lip 1** | fatty acid desaturase 2 | C31404A06 | orange1.1g016781m | GTGGACGAATGGCTGTACCT | ACAAAGGCAATGGTGAGGTC | 184 |
| **Lip 2** | phospholipase D β 1 | C31704G10 | orange1.1g003057m | ATCCGAGCTGCTCAACACTT | TGCAGCAAACCTCTCATTTG | 162 |
| **Def 1** | basic chitinase | C31203F05 | orange1.1g020187m | TAAACCGACTCCCGTACCTG | CAAAAAGGCAGCGATTTCTC | 223 |
| **Def 2** | pathogenesis-related 4 protein | C31208A08 | orange1.1g032389m | TCAACTTGggATGCAAaCAA | GCATTGCCtTTTCCATCAGT | 233 |
| **Act** | actin | CX289161 | orange1.1g037845m | CAGCCATCTCTCATCGGAAT | CCTGTGGACAATGGATGGAC | 329 |
